# Targeting strategies for balanced energy and protein (BEP) supplementation in pregnancy: study protocol for the TARGET-BEP cluster-randomized controlled trial in rural Bangladesh

**DOI:** 10.1186/s13063-024-08135-4

**Published:** 2024-05-13

**Authors:** Eleonor Zavala, Diwakar Mohan, Hasmot Ali, Towfida J. Siddiqua, Rezwanul Haque, Kaniz Ayesha, Khalid Bin Ahsan, Hasan Mahmud Sujan, Nazrana Khaled, Atiya Rahman, Barnali Chakraborty, Brian Dyer, Lee S. F. Wu, Anna Kalbarczyk, Daniel J. Erchick, Andrew L. Thorne-Lyman, Alison Tumilowicz, Kaosar Afsana, Parul Christian

**Affiliations:** 1 Department of International Health, Center for Human Nutrition, Johns Hopkins Bloomberg School of Public Health, Baltimore, USA; 2 The JiVitA Project, Rangpur, Bangladesh; 3 James P. Grant School of Public Health, BRAC University, Dhaka, Bangladesh; 4 The Bill & Melinda Gates Foundation, Seattle, USA; 5 Department of Public Health, North South University, Dhaka, Bangladesh

**Keywords:** Balanced energy and protein supplementation, Maternal nutrition, Gestational weight gain, Birth outcomes, Low birth weight, Small for gestational age, Randomized trial, Bangladesh

## Abstract

**Background:**

The World Health Organization (WHO) recommends balanced energy and protein (BEP) supplementation be provided to all pregnant women living in undernourished populations, usually defined as having a prevalence > 20% of underweight women, to reduce the risk of stillbirths and small-for-gestational-age neonates. Few geographies meet this threshold, however, and a large proportion of undernourished women and those with inadequate gestational weight gain could miss benefiting from BEP. This study compares the effectiveness of individual targeting approaches for supplementation with micronutrient-fortified BEP vs. multiple micronutrient supplements (MMS) alone as control in pregnancy in improving birth outcomes.

**Methods:**

The TARGET-BEP study is a four-arm, cluster-randomized controlled trial conducted in rural northwestern Bangladesh. Eligible participants are married women aged 15–35 years old identified early in pregnancy using a community-wide, monthly, urine-test-based pregnancy detection system. Beginning at 12–14 weeks of gestation, women in the study area comprising 240 predefined sectors are randomly assigned to one of four intervention arms, with sector serving as the unit of randomization. The interventions involving daily supplementation through end of pregnancy are as follows: (1) MMS (control); (2) BEP; (3) targeted BEP for those with pre-pregnancy body mass index (BMI) < 18.5 kg/m^2^ and MMS for others; (4) targeted BEP for those with pre-pregnancy BMI < 18.5 kg/m^2^, MMS for others, and women with inadequate gestational weight gain switched from MMS to BEP until the end of pregnancy. Primary outcomes include birth weight, low birth weight (< 2500 g), and small for gestational age, defined using the 10^th^ percentile of the INTERGROWTH-21st reference, for live-born infants measured within 72 h of birth. Project-hired local female staff visit pregnant women monthly to deliver the assigned supplements, monitor adherence biweekly, and assess weight regularly during pregnancy. Trained data collectors conduct pregnancy outcome assessment and measure newborn anthropometry in the facility or home depending on the place of birth.

**Discussion:**

This study will assess the effectiveness of targeted balanced energy and protein supplementation to improve birth outcomes among pregnant women in rural Bangladesh and similar settings.

**Trial registration:**

ClinicalTrials.gov NCT05576207. Registered on October 5th, 2022.

**Supplementary Information:**

The online version contains supplementary material available at 10.1186/s13063-024-08135-4.

## Introduction

In 2015, an estimated 20.5 million babies, or 14.6%, were born with low birth weight (LBW, < 2500 g), an important predictor of infant mortality, morbidity, poor growth, and development [1, 2]. Over 90% of LBW is estimated to occur in low- and middle-income countries (LMICs), predominantly in South Asia and sub-Saharan Africa [3]. LBW can result from being born prematurely (< 37 weeks gestation) and/or due to intrauterine growth restriction (IUGR) and accounts for 99.5% of the “small vulnerable newborn” types [4, 5]. Small for gestational age (SGA) is an indicator of IUGR and is defined as birth weight by gestational age below the 10th percentile of a reference population [6]. In South Asia, as many as 42% of babies are born SGA, where one in four neonatal deaths is associated with SGA [7, 8]. In addition, infants born SGA face poorer neurodevelopment outcomes [9, 10], and have a greater risk of stunting in childhood [11]. Multiple factors such as short birth intervals [12], maternal infections [13], and young maternal age have been associated with an increased risk of SGA [14]. However, short maternal stature, low pre-pregnancy BMI, low gestational weight gain, and micronutrient deficiencies are independent risk factors for SGA [15–17].

Recently, daily multiple micronutrient supplementation (MMS) containing 15 vitamins and minerals, all at an approximate Recommended Daily Allowance (RDA), has been shown to reduce LBW (RR: 0.88, 95% CI: 0.85–0.91, 18 trials) compared to iron and folic acid (IFA) and moderately reduced the incidence of SGA (RR: 0.92, 95% CI: 0.88–0.97, 17 trials) [18]. In an individual participant data analysis of the trials, MMS showed similar results and also resulted in reduction in preterm birth (RR: 0.92, 95% CI:0.88–0.95) compared to IFA [19]. Due to these benefits, supplementation with MMS is being considered as a replacement for IFA in many countries.

The World Health Organization (WHO) recommends balanced energy and protein (BEP) dietary supplementation be provided to all pregnant women living in undernourished populations to reduce the risk of stillbirths and small-for-gestational-age neonates. Undernourishment is usually defined by a low body mass index (BMI < 18.5 kg/m^2^), where a > 20–39% prevalence of underweight women is considered a high prevalence of underweight, and 40% or higher is considered a very high prevalence [20]. This recommendation is based on a meta-analysis of BEP trials in high-income and LMIC settings, which showed that infants born to supplemented mothers had a significantly reduced risk of stillbirth, increased mean birth weight, and decreased risk of SGA [21]. A comparison of three meta-analyses has demonstrated that among all pregnant women regardless of setting, supplementation effect with BEP on mean birth weight ranges from 41 to 73 g, whereas among undernourished pregnant women or those in LMIC contexts only, the increase in mean birth weight is higher at 66–107 g [21–23].

As BMI rates are increasing globally [24], very few countries meet this criterion at the national level, however; recent analyses demonstrate high variability in nutritional status at subnational levels in LMICs [4]. Further, in 2015, pregnant women living in sub-Saharan Africa and South Asia achieved less than 60% and 64%, respectively, of the recommended gestational weight gain (GWG) in pregnancy [25], a risk factor for LBW, and its underlying causes of SGA and preterm birth (PTB) [26]. Sub-national differences in nutritional status and high rates of inadequate GWG in LMICs stress the need to explore novel targeting strategies for maternal nutrition interventions to address inequities and improve birth outcomes, the evidence for which is lacking [27]. Low pre- or early-pregnancy BMI or low mid-upper arm circumference (MUAC) have been proposed as indicators for identifying nutritionally vulnerable women who could benefit from BEP supplementation [27]. Additionally, regular monitoring of inadequate GWG through antenatal care, combined with counseling and supplementation, could be an effective strategy to improve the course of GWG and mitigate adverse outcomes. However, this combination of strategies has not yet been evaluated in LMIC contexts.

Our research group has been conducting maternal nutrition intervention research in northwestern rural Bangladesh since 2001 [28]. Our large, randomized controlled trial of MMS vs IFA showed 10–12% reduction in LBW, PTB, and stillbirth, although the increase in birth weight was limited at about 50 g and maternal undernutrition was high, with 40% low BMI at baseline [29]. In 2018, the Bangladesh Demographic Health Survey (BDHS) survey reported a national low BMI prevalence of 12% among women of reproductive age, but regional rates ranged from 7.6 to 21.7% [30]. Recently, a new formulation for BEP was proposed through an expert consultation for a ready-to-use BEP, fortified with micronutrients and low-dose calcium and with adequate energy (250–500 kcals) to meet the increased requirements during pregnancy and 14–18 g of high quality protein [31]. This product formulation is being tested in a number of trials in LMICs [32]. Thus, we are conducting a cluster-randomized controlled trial in rural Bangladesh to test the enhanced BEP product versus MMS as standard of care in this study area, both by applying a targeted and untargeted approach to examine their effectiveness on increasing birth weight and reducing SGA. Our hypothesis is that a targeted approach using low BMI and inadequate GWG criteria will result in a higher impact on these birth outcomes compared to BEP supplementation to all pregnant women.

## Methods

The trial protocol was developed in accordance with the Standard Protocol Items: Recommendations for Intervention Trials (SPIRIT) checklist (Additional file 1) and all relevant items from the WHO Trial Registration Data Set are included.

### Study design

**Fig. 1:**
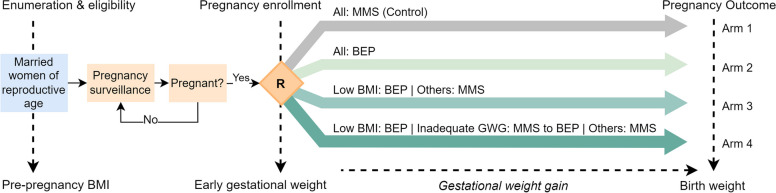
TARGET-BEP study design. BMI, body-mass-index; MMS, multiple-micronutrient supplement; BEP, balanced energy-protein supplement; GWG, gestational weight gain

The TARGET-BEP study is a four-arm, cluster-randomized controlled parallel group 1:1:1:1 superiority trial designed to assess the effect of different strategies for BEP supplementation during pregnancy on the primary outcomes of birth weight, LBW, and SGA in rural Bangladesh (Fig. 1). Newly pregnant women are identified through a pregnancy surveillance system, consented, and enrolled into one of four arms based on the randomized allocation of the cluster in which they reside. From 12 weeks of gestation until pregnancy outcome, participants receive either daily MMS or BEP supplements.

Women in each of the groups receive MMS or BEP supplements, depending on the allocation and targeting criteria, for daily consumption from the time of enrolment, planned at 12 weeks of gestation, until the end of pregnancy (Fig. 1):

- Arm 1 (control): all pregnant women receive daily MMS.
- Arm 2 (all BEP): all pregnant women receive daily BEP supplements.
- Arm 3 (low BMI BEP): pregnant women with a pre-pregnancy BMI < 18.5 kg/m^2^ receive daily BEP supplements, while those with a pre-pregnancy BMI ≥ 18.5 kg/m^2^ receive daily MMS.
- Arm 4 (low BMI and IGWG BEP): pregnant women with a pre-pregnancy BMI < 18.5 kg/m^2^ receive BEP supplements, while others a daily MMS. Among the latter, those identified as having inadequate gestational weight gain (IGWG) are switched from MMS to BEP as described by the protocol below. All other women not experiencing IGWG continue to receive a daily MMS until the end of pregnancy.

Birth outcomes are assessed within 72 h and women and their infants are followed up through 1-month postpartum. Secondary aims include evaluating the effects of each intervention on secondary outcomes of newborn anthropometry and gestation, maternal GWG, and anemia status, and exploring implementation outcomes of acceptability and cost-effectiveness.

### Study setting

The TARGET-BEP study is being conducted in the rural, predominantly agrarian Gaibandha district, Rangpur Division, of northwestern Bangladesh. The climate consists of a dry season from October to April–May, followed by the monsoon season from May to September. In Gaibandha, the diet mainly consists of rice, potato, legumes, seasonal vegetables, and fish products, and dietary diversity has been positively associated with household socioeconomic factors and nutritional status [33]. Micronutrient deficiencies, including anemia, vitamin D, vitamin B-12, and vitamin E, persist in both children and women [34, 35].

**Fig. 2:**
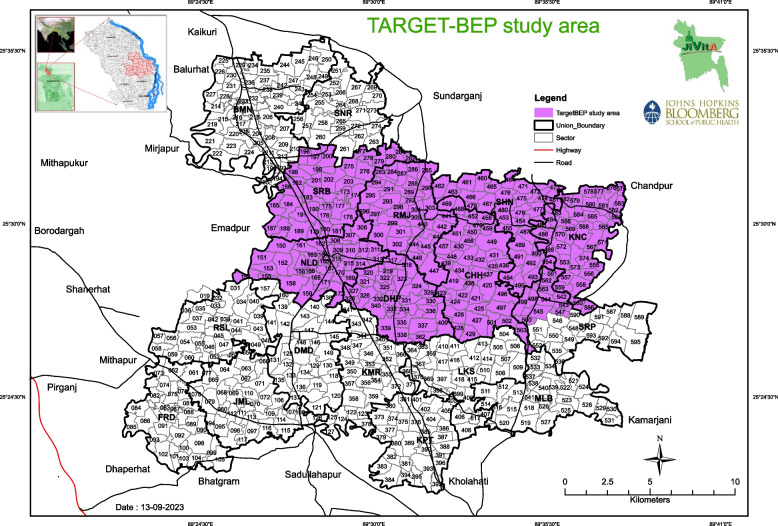
TARGET-BEP study area

The TARGET-BEP study area includes 8 Unions in the Gaibandha district. The study area has been divided into 240 community clusters called sectors which are used as the unit of randomization, each with a number of households ranging from 150 to 200 (Fig. 2). These sectors were drawn at the inception of the JiVitA project two decades ago, and contribute to the larger JiVitA field site, which covers 280km^2^, 284 total sectors, and a total population of women of reproductive age of approximately 75,000.

### Recruitment and eligibility

Recruitment in the study is done using an existing list of married women of reproductive age in the selected sectors. At the outset, a cadre of sector-specific community health research workers (CHRW), local women with high-school degrees or more, employed and trained by the JiVitA project, visited all women to ascertain their current residential and marital status. In addition, a census of all households was done to enumerate women who were newly married or had moved into the study area. Women found in their households are screened for inclusion in a community-based pregnancy surveillance based on the following criteria:

- The woman is 15 to 35 years of age.
- Married and living with husband.
- Neither woman nor husband are permanently sterilized.
- The woman is not currently pregnant and does not have an infant less than one year of age (risk of another pregnancy is low due to lactational amenorrhea and appropriate child spacing).

Consenting participants who meet these criteria have their weight, height, and MUAC measured and are included in a pregnancy surveillance system for home-based visits to ascertain their menstruation history. The pregnancy surveillance system, active throughout the duration of study enrolment, involves the CHRWs visiting the women in their catchment areas every 5 weeks to ask about their last menstrual period (LMP). Participants who report no menstruation in the preceding 30 days during these visits receive a urine-based human chorionic gonadotropin (hCG) test. Pregnant women identified with positive test results are consented for enrolment in the trial, unless they meet one of the exclusion criteria outlined below:

- Women who refuse anthropometry measurements or do not provide consent.
- Women greater than 28 weeks gestation based on their last menstrual period.

To achieve the target sample size, the study area-wide census and enumeration will continue to be implemented every 6 months to allow newly married women or women who have moved into the study area to join the pregnancy surveillance cohort if eligible.

### Consent

Consent in the study is obtained by two cadres of project-hired data collectors. Oral consent for the enumeration and pregnancy surveillance is obtained by CHRWs. Written consent is obtained by a cadre of trained female interviewers (FIs) for enrollment into the trial, and a copy of the signed consent document is provided to the participant. The informed trial consent form includes key information about the study, why the research is being done, what will happen if the participant joins the study, the duration of participation, and what happens to the participant’s data, including clauses on confidentiality and data sharing, the risks and benefits of being in the study, and the IRB contact information to ask questions or register complaints. All consent procedures are administered in the participant’s home, in a private setting and sufficient time is provided to consult with their husband and other senior family members should they wish to.

As part of the main trial consent, we ask participants for their permission to use their de-identified data in future studies. They may choose to refuse that their data be used in ancillary studies and still consent to and participate in the main trial. A point-of-care hemoglobin (Hb) assessment is done to test for anemia, but no biological specimens are collected in the study. Results of the test are provided to the participant.

### Interventions

The BEP product is a packaged, micronutrient fortified ready-to-eat snack in the form of a lipid-based paste, made with milk powder, puffed rice, and lentil or chickpea flour, produced by Frontier Nutrition in Bangladesh (https://frontiernutrition.com/). The nutrient composition follows the expert group consensus specifications [31] and contains 382 kcal, 14.3 g of protein, and 18 micronutrients at 1 RDA for pregnant women per 75 g sachet (Table 1). A formative research study was conducted before the trial to assess the acceptability and short-term adherence of the product among women of reproductive age in the study area [36]. From the findings, minor flavor and processing alterations were made to improve acceptability. Additionally, three product options are offered to women allocated to BEP based on preference: unflavored (lentil-based) and *malai*, a popular clotted-cream flavor found in South Asian cuisine, in either a lentil or chickpea-based format.

**Table 1:** BEP supplement nutritional composition

Nutrients	Per 75 g serving size	Unit
*Macronutrients*		
** Energy**	382.41	Kcal
** Energy from sugar**	39	Kcal
** Fat**	21.81	g
** Protein**	14.30	g
** Carbohydrate**	32.24	g
*Micronutrients*		
** Retinol vitamin A**	770.0	mcg
** Cholecalciferol**	15.0	mcg
** Tocopherol (vitamin E)**	16.0	mg
** Vitamin K1**	90.0	mcg
** Thiamine**	1.40	mg
** Riboflavin (vitamin B2)**	1.40	mg
** Niacin**	18.0	mg
** Pyridoxine (vitamin B6)**	1.90	mg
** Cyanocobalamin (vitamin B12)**	2.60	mcg
** Ascorbic acid (vitamin C)**	100.0	mg
** Calcium**	500.0	mg
** Phosphorus**	383.6	mg
** Copper**	1.00	mg
** Iron**	30.0	mg
** Zinc**	15.0	mg
** Iodine**	220.0	mcg
** Folic acid**	400.0	mcg
** Selenium**	65.0	mcg

The “standard of care” control in the study is a 30-count, blister-packed, daily MMS in a tablet form containing 15 micronutrients at a level of 1 RDA according to the United Nations International Multiple Micronutrient Antenatal Preparation (UNIMMAP) formulation [37]. MMS  was selected as the standard of care comparator given prior evidence of improved birth outcomes and improved maternal micronutrient status compared to IFA in this population [18, 38].

#### Screening criteria

Arm 4 requires BEP supplementation based on IGWG in addition to low pre-pregnancy BMI. In this arm, when a woman is identified with IGWG in the second trimester and for the first time, she receives standard pregnancy nutrition counseling on increasing daily energy and protein intake, as recommended by the WHO antenatal care guidelines [20]. If IGWG is identified again during a future assessment visit, she is switched from MMS to BEP until the end of pregnancy and provided nutrition counseling. If the first IGWG measurement occurs in the third trimester, this triggers a switch from MMS to BEP directly, provided alongside nutrition counseling. Nutrition counseling occurs each time IGWG is detected, regardless of whether the pregnant woman has already been switched to BEP.

We defined IGWG for normal BMI women (18.5–25.0 kg/m^2^) as total weight gain at a given gestational week falling below the 3rd percentile of the INTERGROWTH-21st reference standards [39]. For overweight and obese women (BMI ≥ 25.0 kg/m^2^), IGWG is defined as a rate of weight gain per week below 70% of the recommended rate by the Institute of Medicine (IOM) [40]. We implemented a modification to the IGWG criteria on July 18th, 2023, when approximately 25% of enrollment was completed, based on monitoring the incidence of IGWG in Arm 4. We saw that the proportion of women being switched from MMS to BEP in the normal BMI category was lower than in the overweight/ obese group. This led us to surmise that the 3rd percentile cut-off was too stringent (no guidance about IGWG cut-offs has been provided in the IG-21st studies) and thus we changed the cut-off to be below the 10th percentile of the INTERGROWTH-21st reference standards. At the outset, the cut-off modification resulted in three women in Arm 4 being delayed in switching from MMS to BEP (mean delay 7 weeks), and four women were not switched as their pregnancy outcome had already occurred. Subsequently, all women who met the new cut-off criteria were switched approapriate if found to have IGWG.

#### Intervention delivery and adherence monitoring

In all arms, once a pregnant woman reaches 12 weeks gestation according to her LMP, a CHRW visits her at home to provide a total of 40 daily sachets of BEP or MMS supplements (a month’s supply with 10 extra to not disrupt continuity of supplement use). Home-based delivery of supplements was found to be the preferred method for supplement distribution among community women in the formative study [41]. At the outset, the participant is also provided standard of care nutritional counseling and promotion of antenatal care (ANC) services. She is subsequently followed by monthly home visits to replenish her supplements, until the end of pregnancy. When a woman in Arm 4 meets the criteria described above for switching from MMS to BEP, the CHRW informs her, provides nutrition counseling, and explains to the woman that she will now receive BEP in place of MMS until the end of pregnancy. The final adherence of MMS for such a woman is recorded.

Adherence to supplementation is primarily measured through counting of sachets (BEP) and blister packs (MMS) at the time of the monthly home visit, which are collected back by the CHRW. Additionally, a 2-weekly history of supplement consumption and reasons for non-adherence are collected by a mid-monthly phone call. Special calendars are provided to the women that serve as a visual reminder and to self-record their daily consumption. Messages on the benefits of the supplements, instructions on when and how to consume them, and strategies for reducing sharing and increasing adherence are provided at the time of the first distribution visit, for both BEP and MMS. These messages were designed based on the findings from the formative study [36].

We do not anticipate any adverse reaction or harm related to consuming the MMS or BEP supplement. For any report of intolerance of either supplement, we provide the option to stop supplementation but continue participation in the study or withdraw from the study entirely.

### Outcomes

The primary outcomes of the trial include mean birth weight and proportion of LBW (< 2500 g) and SGA (< 10th percentile of the INTERGROWTH reference standard), among live-born infants measured within 72 h of delivery. Birth weight is measured using the TANITA (BD-585) digital baby scale and gestational age is measured as the time between the date of outcome and the date of last menstrual period (LMP), in weeks. Secondary outcomes are detailed in Table 2 that includ newborn indicators of length-for-age (LAZ) and weight-for-length (WLZ) *Z*-scores and the incidence of large for gestational age (LGA; birth weight for gestational age > 90th percentile), defined using the INTERGROWTH-21st reference standards [42]. The proportion of women with IGWG is defined using the INTERGROWTH-21st reference for normal pre-pregnancy BMI women, as the reference population represents a globally representative group. For the low, overweight, and obese pre-pregnancy BMI women, IGWG is defined using the IOM standards as they are the only available recommendations on GWG for women in these BMI categories [39, 40]. As noted above, IGWG is also an indicator used for screening women in arm 4 for supplementation, as well as a secondary outcome in the study. Weight gain is monitored by the CHRWs with digital adult weighing scales (Taylor 7086), during a home-based visit that is scheduled according to the recommended WHO ANC contacts at 20-, 26-, 30-, 34-, 36-, 38-, and 40-weeks’ gestation.

**Table 2:** TARGET-BEP study secondary outcomes

Newborn	Maternal
1. Mean birth length, head and chest circumference among live-born infants measured within 72 h of delivery	1. Mean maternal weight gain and proportion with inadequate weight gain during pregnancy (using < 10th percentile IG-21st GWG reference for normal BMI women and IOM recommendation for low and high BMI women)
2. Mean length-for-age (LAZ) and weight-for-length *Z*-score (WLZ), and proportion stunted and wasted (< − 2 *Z*-scores) at birth	2. Maternal hemoglobin and anemia (Hb < 11 g/dl) in the third trimester
3. Mean gestational age and proportion preterm (< 37 weeks) among live-born infants	3. Maternal postpartum BMI (1 month)
4. Incidence of large-for-gestational age (LGA) (> 90th percentile of the IG-21st reference standard) among live-born infants measured within 72 h of delivery	4. Maternal dietary intakes in the third trimester among a sub-sample of women

*IG-21st* Intergrowth-21st, *GWG* gestational weight gain, *BMI* body mass index, *IOM* Institute of Medicine

### Sample size

Based on the cluster-randomized design and assuming a sector size of 10 live births per sector with weight measured within 72 h, we estimated requiring (rounded) 60 sectors per arm or a total of 240 sectors to yield 2400 live births, applying a conservative type 1 error of 0.0125 to account for multiple comparisons, and with 80% power to detect an 83 g increase in mean birth weight, a 20% and 24% relative risk (RR) reduction in the incidence of SGA and LBW associated with any of the interventions in the study relative to the control group. The intra-cluster correlation (ICC) was assumed to be 0.005 or less for each of the primary outcomes based on the previous JiVitA-3 trial [29]. To achieve the sample size of 2400 live births and assuming 30% fetal loss and 6% loss to follow-up based on data from the JiVitA-3 trial [29], our enrollment requirement is for approximately 3750 pregnancies, which is planned to take 1.5 years.

### Randomization and allocation

Sectors were randomized in permuted blocks of 8 following a geographically contiguous listing of sectors. The study statistician utilized the Stata 15.0 package “randomizr” to randomly assign the blocked sectors to one of the 4 arms, ensuring balance across arms (24.6%, 25.0%, 25.0%, and 25.4%, respectively). This food-based trial is unblinded and no concealment mechanism for the sector-specific allocation assignment was required and the location of a woman’s household in a sector at the time of enrollment determines her intervention allocation. Following enrollment, the woman is informed of their random assignment by a female interviewer following the informed consent process based on the sector allocation list she is provided. Because each sector is randomized to one of the four arms, all pregnant women residing in that sector receive the same intervention.

Due to the nature of the intervention, it was considered infeasible to mask the participants or study staff to the intervention assignment (unblinded). The BEP arms are easily identifiable using the BEP sachet adherence, and arms 3 and 4 have a variable number of women consuming the product based on an identifiable condition, such as BMI. Additionally, as this is a pragmatic effectiveness trial using an intervention already recommended by WHO given its effect on birth outcomes, we do not plan to mask the analytical team.

### Data collection

**Fig. 3:**
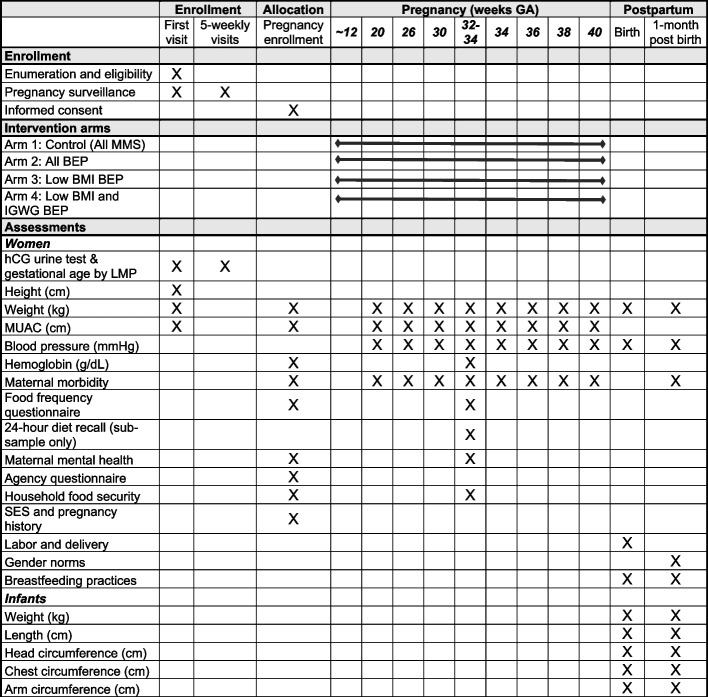
TARGET-BEP trial participant timeline for enrollment, interventions, and assessments. GA, gestational age; MMS, multiple-micronutrient supplement; BEP, balanced energy-protein supplement; IGWG, inadequate gestational weight gain; hCG, human chorionic gonadotropin; LMP, last menstrual period; SES, socioeconomic status

**Fig. 4:**
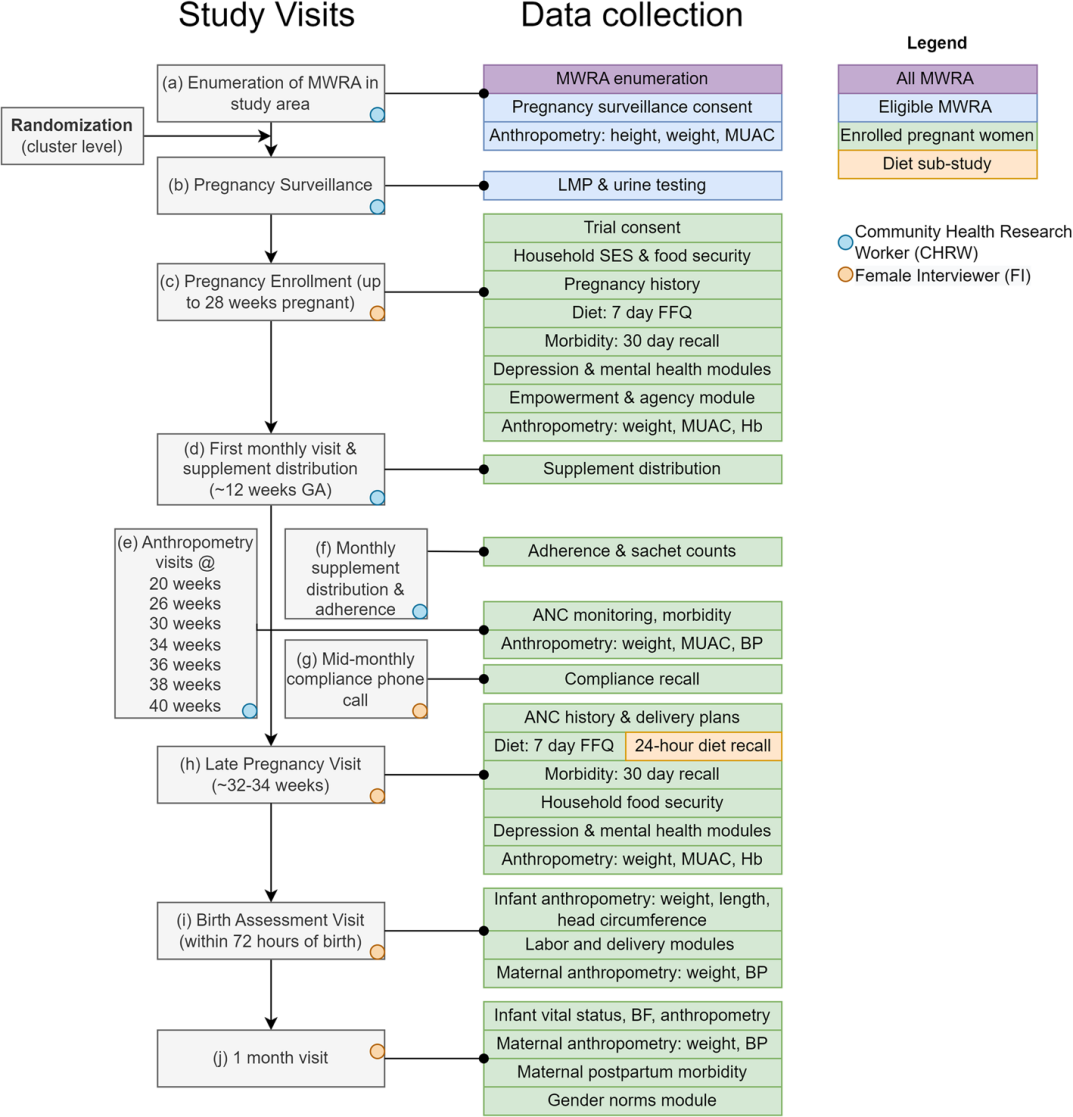
TARGET-BEP study visits, data collection, and procedures. MWRA, married women of reproductive age; MUAC, mid-upper arm circumference; LMP, last menstrual period; SES, socioeconomic status; FFQ, food frequency questionnaire; Hb, hemoglobin; ANC, antenatal care; BP, blood pressure

Figure 3 outlines the participant timeline, including enrollment, interventions, and timing of assessments, and Fig. 4 details the flow of study procedures, including the data collection modules and worker type for each study visit. Following consent, FIs conduct a home-based enrolment interview using a questionnaire, used previously in our trials, to collect demographic and health data on the enrolled woman and her household including household socioeconomic status, pregnancy history, a 7-day food frequency recall of commonly consumed items, 30-day morbidity, 7-day work history and personal hygiene. The Edinburgh Postnatal Depression Scale (EPDS) [43], the Woman’s Agency Scale (WAS) [44], and the Household Food Security Access Scale (HFIAS) are also administered at baseline [45]. Weight, mid-upper arm circumference (MUAC), and hemoglobin assessment using the HemoCue 301 is collected at the time of enrollment. A similar set of assessments, including 30-day morbidity, 7-day food frequency, 7-day work history, personal hygiene, the EPDS, the HFIAS, and anthropometry and hemoglobin are repeated at the late pregnancy visit, occurring between 32 and 34 weeks of gestation. The multiple pass method is used by trained data collectors to conduct a 24-h diet recall assessment in late pregnancy in a sub-sample of 200 women, 50 per arm, to assess whether BEP supplementation results in a replacement of home-based macronutrient intakes. Replicate recalls are collected on non-consecutive days in the same week in 40% of the sample, or 20 per arm.

Using LMP-based gestational age, pregnant women are scheduled for at-home visits at 20-, 26-, 30-, 34-, 36-, 38-, and 40-weeks’ gestation, where weight, MUAC, and blood pressure are measured by their local CHRW. History of ANC services, 7-day morbidity, and consumption of non-study nutritional supplements are also recorded at these visits.

A robust birth notification system supported by the high prevalence of mobile phones and project staff living within the community allows birth assessments to be conducted in a timely manner. Women and their newborns are visited by an FI within 72 hours of birth both at home or in a facility depending on where the birth occurs, with the aim of meeting them within 24 hours, to measure newborn length, weight, MUAC, and head and chest circumference, along with maternal weight and blood pressure. A questionnaire is administered to the mother to collect information on the infant’s vital status and sex, labor and delivery, breastfeeding, and use of pre-lacteals. At 1-month postpartum, the FI revisits the woman to determine the infant’s vital status, repeats the same anthropometry measurements for the infant and mother, collects information on breastfeeding practices and maternal postpartum morbidity, and administers the G-NORM scale to the mother to assess gender norms [46].

The quality of study data is maintained with extensive training of data collectors, regular field observation by supervisors, and everyday monitoring of data entry. All anthropometric measurements are taken in triplicate at each time point, except for weight, which is taken once at each time point. Anthropometry standardization was conducted among all staff before the study started and is repeated every 6 months. Digital weighing scales undergo daily calibration and the HemoCue 301 machines are checked monthly for quality control.

All data collection forms can be found at: https://jivita.org/targetbep/.

In cases where a woman moves out of the study area for delivery, every attempt is made to meet the woman and newborn within a reachable distance to conduct the outcome assessment. Women are considered lost to follow-up if they move out of the study area or refuse to continue participation in study activities. Reasons for withdrawal are recorded.

### Data management

Data collectors use password-protected Android tablets to collect data during household visits via the ONA electronic data collection software. Data collection forms are programmed and tested using the ONA system and data range, plausible values, and other entry restrictions are applied to reduce data entry errors. Forms are assigned on a weekly or daily basis, depending on the timeliness of the data collection (i.e., birth visits) and completion of forms is monitored daily. Data is uploaded and stored on an encrypted server on a weekly basis and subject to data quality checks. Missing or improbable data is investigated by field staff to assure that there are no unusual values, patterns, and entry errors in the datasets.

Datasets prepared for analysis will be de-identified and stored on an encrypted server behind a firewall on an internal network. Access to identifiable information is restricted to the Principal Investigator (PI) and key co-investigators and staff. Only de-identified datasets will be shared beyond the study team.

### Statistical methods

The data analytic plan and approach are briefly described here. A detailed statistical analytic plan is being prepared and will be available in the main trial publication. The unit of analysis for the primary treatment effects analysis will be live births. The primary analysis will be a modified intention-to-treat approach, i.e., all study outcomes will be analyzed as randomized with an analytical sample restricted to those with outcome assessed (birthweight measured within 72 h). We will present crude estimates of all primary and secondary outcomes for each study arm with their 95% confidence intervals adjusted for clustering at the sector level using generalized estimating equations.

Before analyzing treatment effects for primary and secondary outcomes we will assess baseline comparability by allocation arm. All the variables that are assessed at enrollment, i.e., before BEP or MMS provision, will be considered as baseline characteristics, and differences will be examined by comparing values of means, medians, and proportions across arms. Following CONSORT guidelines for effectiveness trials, no statistical tests will be conducted comparing baseline characteristics between study arms but analyses will be both unadjusted and adjusted for variables found to be different across groups.

Continuous outcomes measured at birth (including the primary outcome of birth weight and other anthropometric measurements, gestational age, and maternal Hb in the third trimester) will be estimated using marginal models using linear regression with generalized estimating equations (GEE) to adjust for the cluster-randomization approach used in the trial. For binary outcomes measured at birth (LBW, SGA, PTB, inadequate GWG), we will calculate relative risk ratios (RRRs) and absolute risk differences that will be analyzed using log-binomial regression models with GEE. To control the family-wise error rate, we will use the conservative Bonferroni correction for the type 1 error threshold. Thus, for the four-arm comparisons, the alpha threshold will be 0.0125 to reject the null hypothesis of no difference. In addition to an overall analysis of covariance, each intervention arm will be compared relative to the control as well to each other. Targeted vs. untargeted interventions will also be compared to test whether a targeted approach results in a higher impact (Arm 2 vs. Arms 3 and 4).

If variables are unbalanced at baseline across study arms (defined as more than 2.5% in absolute value), we will assess their associations with the primary and secondary outcomes. If these unbalanced variables are associated with our outcomes, then multivariable analytic approaches will be used to adjust the treatment group differences to control for this imbalance.

Effect modification will be explored for a limited number of covariates collected at enrollment and known to influence birth outcomes. Given that these tests are exploratory in nature, we will assess interaction terms using *p* < 0.1 to determine statistical significance. In the presence of significant effect modification, we will present treatment effects stratified by the covariate(s). The following variables are considered as a priori effect modifiers: (1) BMI < 18.5 kg/m^2^ (underweight status); (2) MUAC < 23 cm; (3) maternal anemia at study inclusion — Hb concentration < 11 g/dL; (4) maternal short stature: height < 145 cm; (5) maternal age < 19 years; (6) parity (nulliparous vs. not); (7) household food insecurity (moderate or severe vs. none, early and later pregnancy). Since adherence to the supplementation is an important covariate of interest, we will measure compliance using different indicators including total packets consumed, mean packets consumed per woman per week, and proportion of women consuming at least 90% and 50% of packets provided. To assess the dose–response, we will analyze the effectiveness estimates between high and low-compliant users based on categories created empirically using median (or tertiles/quartiles/quintiles) values and compare with true controls.

### Oversight and monitoring

#### Data monitoring and auditing

The trial steering committee is led by the PI and is comprised of co-investigators and senior researchers from JHU and BRAC University and the implementing organization, the JiVitA project, including scientists, physicians, a statistician, and a data management specialist. The JiVitA leadership team is responsible for overseeing the day-to-day study activities, including recruitment of participants, obtaining consent, and providing support and monitoring of data collectors and field workers. The trial steering committee meets weekly to monitor study progress and conduct.

Given that we are examining a targeting approach for an evidence-based and recommended intervention by the WHO for BEP supplementation, a Data Safety and Monitoring Board was not deemed necessary. Both the JHU Bloomberg School of Public Health and the BRAC University James P. Grant School of Public Health IRBs provided ethical approval for the study.

#### Adverse events reporting

Serious adverse events (SAE), defined under Good Clinical Practice guidelines, include death, life-threatening events, hospitalization outside of routine delivery, significant or persistent disability or impairment, and congenital anomalies. Data collection staff are trained to systematically identify and report serious and other adverse events (AEs) during regular home visits and research physicians conduct subsequent home visits to determine cause and attribution for SAEs. All SAEs are reported from the field to the PI who is responsible in consultation with the investigative team for assessing relatedness to the intervention and follow the procedures for reporting AEs to the Institutional Review Boards (IRBs). Any maternal and infant deaths, and any unanticipated harms, will be reported in the main trial publication following CONSORT guidelines.

#### Ancillary care

Referral and travel cost to the local health center or hospital is provided for women reporting a group of severe morbidity symptoms and for those with high blood pressure when assessed as part of the interview. Treatment for severe anemia (Hb < 70 g/L) with iron is also provided. All enrolled participants receive an information card that contains the contact information of the IRBs should they wish to report any harm from trial participation.

## Discussion

Undernutrition among women of reproductive age in Bangladesh has reduced in recent years, but regional and sub-regional data show that improvements are not universal, and macro- and micronutrient deficiencies persist [30, 47]. In pregnancy, such deficiencies hinder proper fetal growth and development, contributing to an estimated 23% LBW in Bangladesh nationally [48], which in turn contributes to high mortality and child stunting. In South Asia, 20% of children are stunted at birth and linear growth faltering at birth increases both the risk of stunting later in childhood as well as the risk of stunting relapse among those who recover [49]. While Bangladesh has made substantial progress towards achieving the Sustainable Development Goal target of reducing stunting prevalence to 20% by 2025 [50], further progress may hinge on the success of efforts to address the causes of adverse birth outcomes through effective antenatal care interventions. Micronutrient fortified BEP supplementation, in line with the expert consultation’s recommendations on composition [31], has the potential to address nutritional gaps in pregnancy, when requirements are higher, benefitting maternal health and birth outcomes. A recent trial conducted in Burkina Faso found that supplementation with fortified BEP was associated with improvements in duration of gestation (0.20 weeks, 95% CI: 0.05, 0.36), birth weight (50 g, 95% CI: 8.11, 92.0), and reductions in LBW (− 3.95 percentage points (pp), 95% CI: − 6.83, − 1.06) compared to IFA only [51]. While the main effect for SGA was a 3.1% pp reduction, the confidence intervals were wide (− 7.4, 1.2), suggesting a range of treatment efficacy possibilities. Harmonization of data across five pregnancy trials will provide additional evidence on the effects of enhanced BEP supplementation on birth outcomes [32].

Evidence from previous meta-analyses suggests that undernourished women are more likely to benefit from BEP in terms of birth weight than non-undernourished women [21–23]. With the growing heterogeneity of nutritional status in LMIC contexts, it is imperative to evaluate targeted interventions, which, if effective, would likely have the double benefit of being more equitable and cost-effective [27]. The trial presented in this paper aims to address this research gap. This protocol describes the design of a cluster-randomized trial that will evaluate the effectiveness of two targeting strategies for fortified BEP supplementation, including pre-pregnancy BMI and gestational weight gain monitoring, compared to MMS in pregnancy on birth outcomes. To our knowledge, this is the first trial in Bangladesh to evaluate micronutrient-fortified BEP with and without targeting in pregnancy.

This trial has several strengths. The targeting strategies of pre-pregnancy BMI and gestational weight gain monitoring were developed based on the current literature [27] and in the context of the capacity of the Bangladesh health system. The monthly distribution of supplements represents a realistic distribution schedule for community health workers and was informed by the formative study findings on preference for at home distribution [41]. The BEP product itself was considered highly acceptable by women and health care providers in the formative study [36]. The combination of monthly sachet or blister pack counts and bimonthly participant-reported recall will yield comprehensive adherence data. The timing of anthropometry visits was designed in accordance with the WHO ANC contact schedule, to closely monitor gestational weight gain trajectories and the effect of switching from MMS to BEP. This trial will generate data on several factors influencing pregnancy outcomes, including household socioeconomic status and food security, maternal diet, morbidity and mental health, and women’s agency and gender norms. Finally, the integration of this trial into the long-standing JiVitA project and its infrastructure will ensure the recruitment, follow-up, and outcome assessment of the participants is robust, well-coordinated, and with a low risk of loss to follow-up since the fieldwork team is well-experienced in trial implementation and holds close contact with the local population. Although the study is designed as a cluster-randomized controlled trial, contamination between hamlets or neighborhoods cannot be excluded due to their close-knit location and relationships among the inhabitants. Challenges related to low fertility rates and loss to follow-up due to migration to larger cities being recently observed may impact the study duration and sample size accrual.

Results from the TARGET-BEP trial will provide evidence for targeted BEP supplementation to identify beneficiaries and improve birth outcomes, further enhancing the WHO’s contextual recommendation on BEP supplementation in pregnancy.

## Trial status

Enrollment into the trial began on October 18th, 2022, and is expected to last until May 2024, followed by an additional 7 months of follow-up. As of December 14, 2023, a total of 1880 pregnant women had been enrolled across the four study arms. We are implementing Protocol version 3, on January 5, 2024.

### Supplementary Information

**Additional file 1.** SPIRIT Checklist

## Data Availability

Data will be available upon reasonable request to PI, following guidelines of the BRAC and JHU Institutional Review Boards and local regulations. Following completion of the study, we anticipate taking up to two years to publish primary and secondary outcome papers. Data will be provided to the funder for data-sharing purposes after at least two years of completion of the grant period and according to data-sharing agreements with collaborators. Participant-level data when shared will be deidentified. The statistical code can be provided upon request.

## Abbreviations

WHO: World Health Organization
BEP: Balanced energy protein
MMS: Multiple micronutrient supplements
BMI: Body mass index
LBW: Low birth weight
LMIC: Low- and middle-income countries
IUGR: Intrauterine growth restriction
SGA: Small for gestational age
RDA: Recommended Dietary Allowance
IFA: Iron and folic acid
GWG: Gestational weight gain
PTB: Preterm birth
MUAC: Mid-upper arm circumference
BDHS: Bangladesh Demographic Health Survey
SPIRIT: Standard Protocol Items: Recommendations for Intervention Trials
CHRW: Community health research worker
LMP: Last menstrual period
hCG: Human chorionic gonadotropin
FI: Female interviewer
Hb: Hemoglobin
UNIMMAP: United Nations International Multiple Micronutrient Antenatal Preparation
IGWG: Inadequate gestational weight gain
IOM: Institute of Medicine
ANC: Antenatal care
LAZ: Length-for-age *z*-score
WLZ: Weight-for-length *z*-scores
LGA: Large for gestational age
RR: Relative risk
EPDS: Edinburgh Postnatal Depression Scale
WAS: Women’s Agency Scale
HFIAS: Household Food Security Access Scale
PI: Principal Investigator
SAE: Serious adverse event
AE: Adverse event
IRB: Institutional Review Board
